# Effects of dehydration and blockade of angiotensin II AT1 receptor on stress hormones and anti-oxidants in the one-humped camel

**DOI:** 10.1186/1746-6148-9-232

**Published:** 2013-11-19

**Authors:** Mahmoud Alhaj Ali, Elsadig Kazzam, Naheed Amir, Fred Nyberg, Abdu Adem

**Affiliations:** 1 Department of Pharmacology and Therapeutics, Faculty of Medicine and Health Sciences, United Arab Emirates University, P.O. Box 17666, Al Ain, United Arab Emirates; 2 Department of Pharmacology and Therapeutics, Faculty of Pharmacy, Uppsala University, Uppsala, Sweden; 3 Department of Internal Medicine, College of Medicine and Health Science, United Arab Emirates University, Al Ain, United Arab Emirates

**Keywords:** Camel, Catecholamine, Cortisol, Dehydration, Glutathione, Losartan and malondialdehyde

## Abstract

**Background:**

The objective of this study was to provide for the first time data on plasma catecholamines, cortisol, glutathione and malondialdehyde after long term dehydration (20 days) in the presence and absence of angiotensin II (Ang II) AT1 receptor blocker (losartan) versus levels in time-matched, non-dehydrated control camels and to record the responses of glutathione and malondialdehyde activity in liver and kidney homogenates in control, dehydrated-losartan treated and dehydrated camels. Eighteen male camels were studied, six hydrated (control group), six dehydrated and treated with losartan (treated group) and six dehydrated not treated (dehydrated).

**Results:**

Plasma levels of norepinephrine and dopamine were significantly increased (P < 0.01) in both treated and dehydrated groups compared to time matched control, whereas Plasma epinephrine level showed significant decrease (P < 0.05) in both treated and dehydrated groups compared to control. Plasma cortisol also showed significant increase (P < 0.01) in both treated and dehydrated groups compared to control. Glutathione levels in plasma, liver and kidney homogenates for both treated and dehydrated groups reveled significant increase (P < 0.05) Likewise, malondialdehyde levels in plasma, liver and kidney homogenates were substantially and significantly increased in both treated and dehydrated groups.

**Conclusion:**

In conclusion, the results of this study demonstrated that dehydration substantially increased the circulating levels of norepinephrine, dopamine and cortisol but decreased plasma epinephrine. Similarly, losartan showed similar effects to that of dehydration. In addition, this investigation showed dehydration alone or in combination with losartan induced significant increments in glutathione and malondialdehyde activities in plasma, liver and kidney homogenates, presumably in order to counteract the potentially damaging effects of free radicals. Blockade of angiotensin II AT1 receptors did not alter significantly the response of dehydration in any of these indices.

## Background

The maintenance of body fluids is dependent upon the balance between the ingestion and excretion of fluids and solute with any changes in this balance being sensed by the hypothalamic osmoreceptors which regulate the secretion of anti-diuretic hormone (ADH) and thereby modulating the renal excretion of water [1]. The effect of dehydration on some of these hormones is well documented in different species. Dehydration in the camel activates the renin-angiotensin system(RAS) inducing substantial increments in serum sodium, creatinine, urea, and arginine vasopressin [2]. Circulating Angiotensin, derived from the renin angiotensin system is known to influence the sympathetic nerve activity at multiple levels including the central neuraxis, the ganglia and the peripheral sympathetic nerve terminals [3]. Angiotensin 11 (Ang II) acts through two receptor subtypes, AT1 and AT2 receptors that are cloned and characterized pharmacologically [4,5]. Most of the classic physiological effects of Ang II, such as sodium and water retention, vasoconstriction, and aldosterone and vasopressin release are mediated by the AT1 receptor [6]. With the advent of drugs such as losartan which specifically block the Ang II AT-1 receptor, it is possible to dissect the physiological role of the RAS under various circumstances including dehydration. Dehydration is associated with activation of hormonal systems as RAS, aldosterone and antidiuretic hormone [2] which serve to maintain arterial pressure and retain sodium and water, and suppression of the atrial natriuretic peptides (ANP) and B-type natriuretic peptide (BNP), which are vasodilator, natriuretic and diuretic [7]. Although it has been reported that dehydration activates the sympathetic nervous system in some species [8,9] we are not aware that this has been documented in the camel. Accordingly, we investigated the effect of sustained dehydration on circulating indices of sympathetic nervous system activity and plasma cortisol in the camel. Catecholamines are hormones and neurotransmitters in the sympatho-adrenal nervous system produced by the adrenal glands. They are released into the blood circulatory system during physical or emotional stress. The major catecholamines are dopamine (DA), norepinephrine (NE) and epinephrine (EP). Epinephrine causes increase in heart rate, contraction of blood vessels, and dilatation of the respiratory air passages [10]. Norepinephrine is secreted by certain nerve endings of the sympathetic nervous system, and by the medulla of the adrenal glands. Its primary function is to help maintain a constant blood pressure by stimulating certain blood vessels to constrict when the blood pressure falls below normal. Catecholamines are considered as an integral part of the homeostatic response to various stresses in the body and produce vasoconstriction and raise blood pressure, so their concentration increases largely after heat stress and hypothermia [11]. Cortisol is a corticosteroid hormone produced by the adrenal cortex. It is also considered as one of the stress hormones. Although, the primary function of cortisol is to increase blood sugar in the liver as glycogen, it plays an important role on carbohydrate, protein and lipid metabolism and helps in immune function [12]. It had been reported that injection of cortisol induced diuresis leading to dehydration in ruminants under heat stress [13]. Dehydration was mentioned to cause oxidative damage to body cells by either random formation of intermolecular disulphide bridges, or by uncontrolled oxidation of thiols to sulphonic acids [14]. Dehydration with exercise increased circulating cortisol levels in healthy humans [15] although the contrary, a decline in plasma cortisol, was documented during water restriction in pregnant mares [16].

Glutathione (GSH) is a simple molecule, produced naturally by the combination of three amino acids–cysteine, glycine and glutamine. It is known that GSH is produced by cells to neutralize the free radicals and reactive oxygen compounds and is essential for the immune system to exert its full potential in proliferation of lymphocytes [17]. GSH as cellular Anti-oxidant can protect thiol-containg enzymes and can directly scavenge free radicals [18]. Malondialdehyde (MDA) is a naturally occurring endogenous product of lipid peroxidation and prostaglandin biosynthesis. The production of MDA is used as a biological marker of endogenous DNA damage [19]. In this study, we documented the responses of the sympathetic nervous system and cortisol in dehydrated camels with and without blockade of AngII AT1 receptors. We also measured GSH and MDA levels in plasma, liver and kidney homogenates in control and dehydrated with or without blockade of AngII AT1 receptors in the one-humped camel.

Our hypothesis was that: dehydration would activate stress hormones, leading to increased levels of plasma catecholamines and cortisol levels; in addition GSH and MDA levels would be elevated in plasma as well as in liver and kidney homogenates. Moreover, blockade of AngII AT1 receptors with losartan would attenuate the responses of catecholamines and cortisol to dehydration and inhibit GSH and MDA levels in plasma and tissue homogenates.

### Ethical approval

The study protocol was approved by the Animal Ethics Committee of the United Arab Emirates University. The approval ID was AE/03/38. The camels were visited daily by an experienced Veterinarian to ensure their well-being.

## Methods

### Animals used

Eighteen male camels, aged 3-4 years were studied. The animals were divided into three groups: six hydrated or control group, six dehydrated and losartan-treated group and six dehydrated. The control group was given food and water ad libitum while the treated and dehydrated groups were given food ad libitum but underwent water deprivation for 20 days. In addition, the treated group was injected intra-venously with Ang II AT1 receptor blocker (losartan 5 mg/kg/daily for 20 days). All camels were maintained on dry hay and freshly cut green grass.

### Collection of blood samples

Blood was collected from the external jugular vein between 0800 and 1000 hours from all three groups at the same time. Blood for the measurement of catecholamines, cortisol, GSH and MDA was collected into K3-EDTA vacutainers (with aprotonin 500 KIU/ml of blood) on ice, centrifuged at +4°C within one hour after collection and the plasma stored as aliquots at-80°C until analyzed. Liver and kidney tissues were collected in dry ice immediately after slaughtering the animals at the end of the experiment and stored at-800°C until analyzed.

### High performance liquid chromatography (HPLC) Catecholamines

Plasma catecholamines extraction was done using HPLC [20]. Briefly, 2 ml of plasma was added to 15 mg acid washed alumina and 1.0 ml Tris buffer (1.5 M, & 8.5pH), vortexed and mixed for 5 min in a rotary mixture. The alumina was separated and washed twice with double distilled water. After centrifugation the catecholamines eluted with 100 ul of 0.1 M acetic acid, then 50 ul of this solution injected into the system.

### Radioimmunoassay technique

Plasma cortisol together was measured by (RIA) radioimmunoassay technique [2]. Briefly, cortisol was extracted from plasma samples onto a Sep-Pak C18 columns pre-equilibrated with 1% trifluoroacetic acid, then eluted with 60% acetonitrile in 1% trifluoroacetic acid and evaporated to dryness. The residue was reconstituted in assay buffer and cortisol-immunoreactivity measured using a specific radioimmunoassay kit (Peninsula Laboratories, CA, USA). The Inter assay coefficient of variation for cortisol was 7%.

### Colorimetric assay

Plasma GSH and MDA levels were measured by colorimetric assay kits. Tissue samples collected from all the three groups were homogenized using a polyteron homogenizer (IKA laboratory, Germany). The measurement of GSH used a kinetic assay in which catalytic amounts (nmoles) of GSH cause a continuous reduction of 5,5¢-dithiobis (2-nitrobenzoic acid) (DTNB) to TNB and the glutathione disulfide (GSSG) formed was recycled by glutathione reductase and NADPH. The yellow color product, 5-thio-2-nitrobenzoic acid (TNB) was measured spectrophotometrically at 412 nm. MDA level for all groups was measured using MDA Assay kit (Northwest Life Science Specialties, LLC 16420 SE McGillivray, Suite 103, PMB 106, Vancouver, WA 98683, USA). In brief, 250 μl of deproteinated tissue sample was added to 250 μl of 1 M phosphoric acid and 250 μl of butylated hydroxytoluene in ethanol, and then the mixture was heated at 600C for 60 min. The suspension was cooled to room temperature, centrifuged at 10,000 g for 3 min and the pink colored supernatant was taken for spectroscopic measurements at 532 nm for the assay of MDA. Inter assay variation for was GSH 4.5% and for MDA was 2.4%.

### Statistical analysis

The recorded values for all groups were expressed as Mean ± SEM. Differences between the 3 groups of camels were determined by one-way ANOVA using SPSS version 19. Statistical significance was assumed at P < 0.05.

## Results

**Figure 1:**
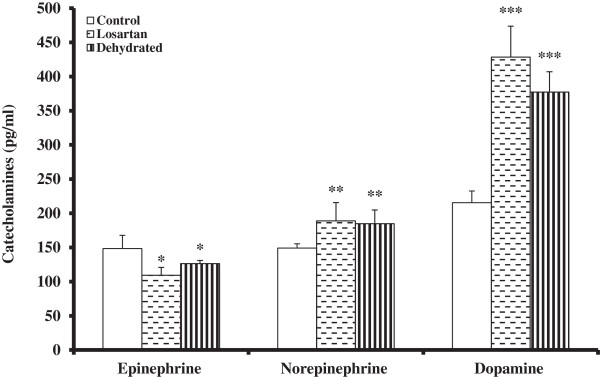
**Catecholamines concentration (pg/ml) in plasma of control, treated and dehydrated camels.** Plasma cortisol level in treated and dehydrated groups was shown as percentage of controls.

Plasma epinephrine levels did not change significantly over 20 days in control group (148.31 ± 19.35 pg/mL), but decreased significantly (p < 0.01) after 20 days of dehydration in both treated and dehydrated groups compared to time-matched control. The overall decrease was greater in the treated group (109.36 ± 11.59 pg/mL) than in the dehydrated group (126.38 ± 4.78 pg/mL), albeit, no significant difference was seen between the two dehydrated groups. Plasma norepinephrine level remained steady in the control group (149.122 ± 6.10 pg/mL), whereas both treated and dehydrated groups showed significant increase (p < 0.01). Plasma dopamine level was unaltered after 20 days in control camels while both treated and dehydrated camels exhibited statistically significant increase (p < 0.001). The increase in dopamine levels was higher in the treated group, but without any significant differences from the dehydrated group (Figure 1).

**Figure 2:**
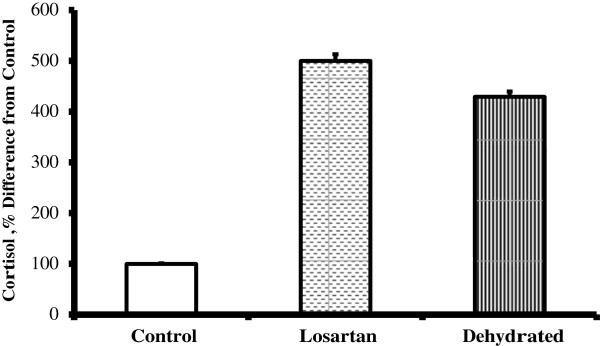
**Cortisol concentrations (μg/dl) in plasma of control, treated and dehydrated camels.** Data shown are mean ± SEM. Asterisks denote significance (** P < 0.01) from control.

Plasma cortisol levels in control camels (0.17 ± 0.1 μg/ml) did not change significantly during the 20 day study. By contrast cortisol level increased approximately 3-fold in both treated (0.85 ± 0.131 μg/ml) and the dehydrated groups (0.73 ± 0.13 μg/ml). No significant difference between the two dehydrated groups was seen (Figure 2). Plasma cortisol level in the treated and dehydrated groups was shown as percentage of control in Figure 2.

**Figure 3:**
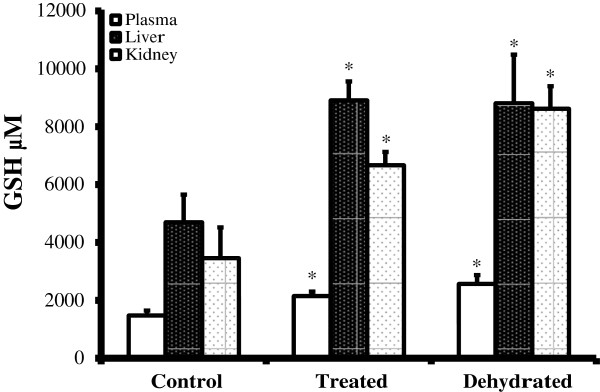
**GSH activities (μM/ml) in plasma, liver and kidney homogenates of control, treated and dehydrated camels.** Data are mean ± SEM. Asterisk denotes significance from control (*P < 0.05).

Plasma GSH level in control camels was 1479.99 ± 167.28 μm/l on day 20. The effect of dehydration alone caused a significant increase in glutathione level to 2570.35 ± 299.34 μm/l. while, the combined effects of dehydration and losartan caused glutathione level to increase, but to a lesser extent than that of dehydration alone 2151.35 ±146.68 μm/l (Figure 3). GSH concentration in liver homogenate for the control camels was 4699.04 ± 809.06 μm/l, and that for both the treated and the dehydrated camels were significantly increased to 8915.99 ± 686.24 and 8814.09 ± 844.85 μm/l respectively. GSH level in kidney homogenate was 2959.01 ± 119.06 μm/l for control camels and for the treated and dehydrated was two and three folds (6670.02 ± 454.86 and 8623.18 ± 774.97 μm/l respectively) (Figure 3).

**Figure 4:**
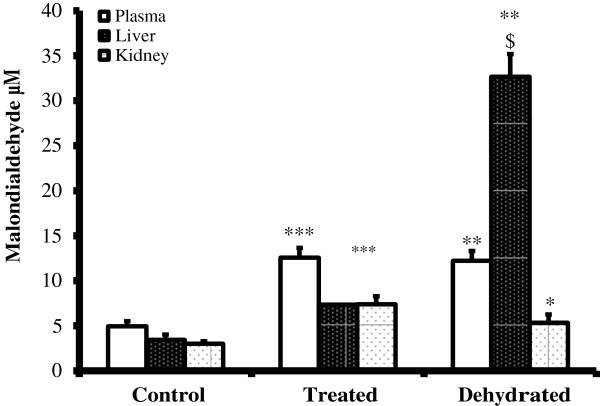
**Malondialdehyde activities (μM/ml) in plasma, liver and kidney homogenates of control, treated and dehydrated camels.** Data are mean ± SEM. Asterisk denotes significance from control (* P < 0.05, ** P < 0.01).

Plasma malondialdehyde level in control camels was 4.95 ± 0.56 μm/l. The level in both dehydrated groups increased significantly (P < 0.001) after 20 days of water deprivation in presence of losartan (12.58 ± 1.07 um/l) and in absence of losartan (12.23 + 1.07 um/l) compared to control camels (Figure 4). MDA level in liver homogenate in control was 3.46 ± 0.56 μm/l. In the treated the level was doubled (7.33 ± 1.83 μm/l) (but this result was from one camel only and was not used for comparison and statistical significance). On the other hand, the dehydrated group liver homogenate showed significant (P < 0.01) increase, reaching (10 folds compared to control). Results from kidney homogenate for control was 3.02 ±0.26 μm/l and for the treated was 7.04 ± 0.88 μm/l, while for the dehydrated was 5.35 ± 0.91 μm/l (Figure 4).

## Discussion

A large body of evidence suggests that the RAS system interacts with the sympathetic nervous system [21,22,23]. Angiotensin II (Ang II) has been shown to increase vascular sensitivity to norepinephrine in vivo as well as in isolated vessels [21] leading to the suggestion that Ang II and norepinephrine exert synergistic actions on vascular tone. It has also been reported that blockade of endogenous Ang II by AT1 blockers could alter vascular reactivity to exogenous norepinephrine [22,23]. Moreover, Ang II is well known to facilitate sympathetic influences on the systemic circulation. Catecholamines as neurotransmitters and adrenomedullary hormones influence almost all tissues and many body functions. Together with other neuronal and hormonal systems they play a significant role in regulating a number of physiological processes. Our results demonstrated that dehydration activated some components of the sympathetic nervous system as reflected in the circulating levels of norepinephrine and dopamine. Blockade of AngII AT1 receptors tended to exaggerate the effects of dehydration, although differences from dehydration alone failed to reach levels of statistical significance. In this study we found an increased plasma norepinephrine levels for treated and dehydrated camels, at the same time epinephrine level in both treated and dehydrated camels was reduced compared to time matched control camels, the reason for that could be due to the stress effects of dehydration with or without losartan. It has been mentioned in similar studies of norepinephrine and epinephrine under the stress of hemorrhage and dehydration in frogs showed that the higher tolerance to hemorrhage and dehydration could be in part due to the greater adrenal development and activity [24]. It had been mentioned that plasma epinephrine and corticotrophin responses to hypoglycemia are larger than norepinephrine responses and that plasma norepinephrine response to cold exposure was larger than epinephrine and corticotrophin responses [25]. Others have reported that different stress factors can produce different responses in catecholamine concentrations, but the exact details of the activation mechanism of catecholaminergic systems under various stressors is not well understood [26]. The increase in plasma catecholamine levels was correlated with an increase in vascular resistance and that dehydration will cause an increase in plasma catecholamine levels when body mass is reduced by 15-20% due to dehydration [21]. In earlier publication we demonstrated that the body weight of our treated group decreased by 39.1% across dehydration whereas the reduction in body weight for the dehydrated group was 34.5% compared to controls [22]. Therefore, the increased level of plasma norepinephrine in the treated and dehydrated camels could be due to the physical stress caused by dehydration alone or in combination with the effect of losartan. The increased levels of norepinephrine in these camels may have played a positive role in the regulation of their cardiovascular and metabolic systems. Recently it was reported that hormones released during stressful conditions are used to supply the body with the energy required for breaking down the body metabolites [23]. On the other hand, plasma dopamine level was significantly increased after 20 days of dehydration in both treated and dehydrated groups compared to time matched control. Dopamine was reported to regulate fluid and electrolyte balance by direct and indirect actions in the kidney, blood vessels, gastrointestinal tract, adrenal glands, sympathetic nervous system, hypothalamus and other brain centers [27].

Another study reported that three days of water deprivation caused an increase in hematocrit and plasma sodium concentration together with the increase of dopamine accumulation in the posterior pituitary [28]. Dopamine exhibited higher response to the dehydration stress with and without losartan, suggesting that dopamine plays an important role in these dehydrated camels. Plasma cortisol levels increased during dehydration to more than 2-fold for the dehydrated and 3-fold for the treated group when compared with time-matched control. The increase in cortisol level was higher in the treated versus dehydrated camels, albeit, no significant difference between them was observed. An earlier study in camels showed no change in plasma cortisol during dehydration [29], but the small number of animals studied (n = 3), the relatively brief duration of dehydration (10 days) and the absence of non-dehydrated control hampers the interpretation of that data. The main cause for the vigorous rise in plasma cortisol in these dehydrated camels is not immediately apparent. The most obvious possibility is that the hypothalamic-pituitary axis was stimulated during dehydration resulting in a rise in plasma adrenocorticotropic hormone (ACTH) and thence cortisol. This could be the result of “stress” or possibly due to activation of RAS since AngII, at least under some circumstances, can stimulate or enhance the release of corticotropin-releasing factor [30] and ACTH [31,32] and, in addition, is capable of stimulating cortisol secretion directly from the adrenal fasciculata [33,34] or augmenting the effect of ACTH on cortisol production [35]. It had been reported that cortisol had the capacity to interfere with a principal mechanism of resistance to dehydration, by inhibiting the effects of arginine vasopressin in dogs [36]. Other studies showed that effect of external heat load could be associated with significant increments of plasma cortisol [37].

Dehydration enhanced by losartan activated body antioxidants resulting in a significant increase in plasma GSH levels after 20 days of dehydration compared to time-matched control. GSH level in liver homogenate was also significantly increase in both treated and dehydrated groups when compared with control. However, GSH level in liver homogenate was more affected by the presence of losartan than with dehydration alone. The liver is known to have the highest content of antioxidant and antioxidant enzymes indicating that it plays an important role in pro-oxidants detoxification [38]. GSH level in kidney homogenate was higher in both treated and dehydrated camels compared to time-matched control. Dehydration is known to cause an increase in production of reactive oxygen species that leads to lipid peroxidation and nucleic acid damage causing severe consequences on over all metabolism [39]. Thus, the increase in GSH level in plasma and liver homogenate in the treated and dehydrated camels indicate that there is an increased level of oxidative stress in these dehydrated camels. This can explain the increased levels of GSH in both the treated and dehydrated camels that might play an important role in alleviating the devastating effect caused by the reactive oxygen species produced by dehydration and losartan.

The present study also revealed higher increase in MDA concentrations in plasma, liver and kidney homogenates in both treated and dehydrated camels compared to control. It had been reported that increased production or decreased removal of hydrogen peroxide lead to lipid peroxidation and this results in an increased production of plasma MDA level [36]. It was reported that dehydration causes high production of reactive oxygen species that trigger high lipid peroxidation resulting in an increased MDA formation [35]. Analysis of kidney homogenate also revealed higher increase in MDA level for both treated and dehydrated camels compared to time matched control.

Some limitations inherent in our study need to be mentioned. First, we used circulating levels of catecholamines as an index of global sympathetic activity whereas these levels reflect not only their rate of production but also their rate of clearance from the circulation. Furthermore, venous plasma norepinephrine levels give no clue as to the tissues or organs of origin. Second, we have presumed that changes in cortisol levels reflected concomitant changes in ACTH; we did not confirm this with measurements of plasma ACTH. Third, interpretation of our results would have benefited from measurements of arterial pressure but such measurements are particularly difficult over prolonged periods in the camel. For example had we shown that losartan administration across dehydration induced a substantial decline in arterial pressure compared with dehydration alone, this may have provided sufficient stimulation of the sympathetic nervous system and the Corticotropin Releasing Factor (CRF) /ACTH/cortisol system to have overcome any inhibitory effect from blockade of AngII AT1 receptors by losartan.

## Conclusion

In conclusion, the results of this study demonstrated that dehydration substantially increased the circulating levels of norepinephrine, dopamine and cortisol but decreased plasma epinephrine. Moreover, blockade of angiotensin II AT1 receptors showed similar effects to that of dehydration. In addition, this investigation showed dehydration alone or in combination with angiotensin II AT1 receptor blocker induced significant increments in glutathione and malondialdehyde activities in plasma, liver and kidney homogenates, presumably in order to counteract the potentially damaging effects of free radicals. Blockade of angiotensin II AT1 receptors did not alter significantly the response of dehydration in any of these indices.

## Competing interest

There is no conflict of interest that could be perceived as prejudicing the impartiality of the research reported.

## Authors’ contributions

MA Ali oversaw the day-to-day running of the study (caring for and feeding the animals, collecting and delivering blood samples, measuring body weights and collecting data for analysis) and also contributed to writing the paper. AA planned and advised on conduct of the study, contributed and assembled all data, provided statistical analysis, and contributed to writing of the manuscript. NA performed laboratory analysis of HPLC, Radioimmunoassay technique and Colorimetric assay; oversaw quality control of the different assays. FN carried out all studies of angiotensin receptors in liver tissue and provided general over sight of the whole study. EK conceived of the study and provided general over sight of its conduct. He constructed first draft and over-saw its completion. All authors read and approved the final manuscript.
